# The trend of disruption in the functional brain network topology of Alzheimer’s disease

**DOI:** 10.1038/s41598-022-18987-y

**Published:** 2022-09-02

**Authors:** Alireza Fathian, Yousef Jamali, Mohammad Reza Raoufy, for the Alzheimer’s Disease Neuroimaging Initiative, Michael W. Weiner, Norbert Schuf, Howard J. Rosen, Bruce L. Miller, Thomas Neylan, Jacqueline Hayes, Shannon Finley, Paul Aisen, Zaven Khachaturian, Ronald G. Thomas, Michael Donohue, Sarah Walter, Devon Gessert, Tamie Sather, Gus Jiminez, Leon Thal, James Brewer, Helen Vanderswag, Adam Fleisher, Melissa Davis, Rosemary Morrison, Ronald Petersen, Cliford R. Jack, Matthew Bernstein, Bret Borowski, Jef Gunter, Matt Senjem, Prashanthi Vemuri, David Jones, Kejal Kantarci, Chad Ward, Sara S. Mason, Colleen S. Albers, David Knopman, Kris Johnson, William Jagust, Susan Landau, John Q. Trojanowki, Leslie M. Shaw, Virginia Lee, Magdalena Korecka, Michal Figurski, Steven E. Arnold, Jason H. Karlawish, David Wolk, Arthur W. Toga, Karen Crawford, Scott Neu, Lon S. Schneider, Sonia Pawluczyk, Mauricio Beccera, Liberty Teodoro, Bryan M. Spann, Laurel Beckett, Danielle Harvey, Evan Fletcher, Owen Carmichael, John Olichney, Charles DeCarli, Robert C. Green, Reisa A. Sperling, Keith A. Johnson, Gad Marshall, Meghan Frey, Barton Lane, Allyson Rosen, Jared Tinklenberg, Andrew J. Saykin, Tatiana M. Foroud, Li Shen, Kelley Faber, Sungeun Kim, Kwangsik Nho, Martin R. Farlow, AnnMarie Hake, Brandy R. Matthews, Scott Herring, Cynthia Hunt, John Morris, Marc Raichle, Davie Holtzman, Nigel J. Cairns, Erin Householder, Lisa Taylor-Reinwald, Beau Ances, Maria Carroll, Sue Leon, Mark A. Mintun, Stacy Schneider, Angela Oliver, Lisa Raudin, Greg Sorensen, Lew Kuller, Chet Mathis, Oscar L. Lopez, MaryAnn Oakley, Steven Paul, Norman Relkin, Gloria Chaing, Lisa Raudin, Peter Davies, Howard Fillit, Franz Hefti, M. Marcel Mesulam, Diana Kerwin, Marek-Marsel Mesulam, Kristine Lipowski, Chuang-Kuo Wu, Nancy Johnson, Jordan Grafman, William Potter, Peter Snyder, Adam Schwartz, Tom Montine, Elaine R. Peskind, Nick Fox, Paul Thompson, Liana Apostolova, Kathleen Tingus, Ellen Woo, Daniel H. S. Silverman, Po H. Lu, George Bartzokis, Robert A. Koeppe, Judith L. Heidebrink, Joanne L. Lord, Steven G. Potkin, Adrian Preda, Dana Nguyenv, Norm Foster, Eric M. Reiman, Kewei Chen, Adam Fleisher, Pierre Tariot, Stephanie Reeder, Steven Potkin, Ruth A. Mulnard, Gaby Thai, Catherine Mc-Adams-Ortiz, Neil Buckholtz, John Hsiao, Marylyn Albert, Marilyn Albert, Chiadi Onyike, Daniel D’Agostino, Stephanie Kielb, Donna M. Simpson, Richard Frank, Jefrey Kaye, Joseph Quinn, Betty Lind, Raina Carter, Sara Dolen, Rachelle S. Doody, Javier Villanueva-Meyer, Munir Chowdhury, Susan Rountree, Mimi Dang, Yaakov Stern, Lawrence S. Honig, Karen L. Bell, Daniel Marson, Randall Grifth, David Clark, David Geldmacher, John Brockington, Erik Roberson, Hillel Grossman, Efe Mitsis, Leyla de Toledo-Morrell, Raj C. Shah, Debra Fleischman, Konstantinos Arfanakis, Ranjan Duara, Daniel Varon, Maria T. Greig, Peggy Roberts, James E. Galvin, Brittany Cerbone, Christina A. Michel, Henry Rusinek, Mony J. de Leon, Lidia Glodzik, Susan De Santi, P. Murali Doraiswamy, Jefrey R. Petrella, Terence Z. Wong, Olga James, Charles D. Smith, Greg Jicha, Peter Hardy, Partha Sinha, Elizabeth Oates, Gary Conrad, Anton P. Porsteinsson, Bonnie S. Goldstein, Kim Martin, Kelly M. Makino, M. Saleem Ismail, Connie Brand, Kyle Womack, Dana Mathews, Mary Quiceno, Ramon Diaz-Arrastia, Richard King, Myron Weiner, Kristen Martin-Cook, Michael DeVous, Allan I. Levey, James J. Lah, Janet S. Cellar, Jefrey M. Burns, Heather S. Anderson, Russell H. Swerdlow, Neill R. Graf-Radford, Francine Parftt, Tracy Kendall, Heather Johnson, Christopher H. van Dyck, Richard E. Carson, Martha G. MacAvoy, Howard Chertkow, Howard Bergman, Chris Hosein, Sandra Black, Bojana Stefanovic, Curtis Caldwell, Ging-Yuek Robin Hsiung, Howard Feldman, Benita Mudge, Michele Assaly, Andrew Kertesz, John Rogers, Charles Bernick, Donna Munic, Andrew Kertesz, Andrew Kertesz, John Rogers, Elizabether Finger, Stephen Pasternak, Irina Rachinsky, Dick Drost, Carl Sadowsky, Walter Martinez, Teresa Villena, Raymond Scott Turner, Kathleen Johnson, Brigid Reynolds, Marwan N. Sabbagh, Christine M. Belden, Sandra A. Jacobson, Sherye A. Sirrel, Neil Kowall, Ronald Killiany, Andrew E. Budson, Alexander Norbash, Patricia Lynn Johnson, Joanne Allard, Alan Lerner, Paula Ogrocki, Leon Hudson, Smita Kittur, Michael Borrie, T-Y Lee, Rob Bartha, Sterling Johnson, Sanjay Asthana, Cynthia M. Carlsson, J. Jay Fruehling, Sandra Harding, Vernice Bates, Horacio Capote, Michelle Rainka, Douglas W. Scharre, Maria Kataki, Anahita Adeli, Eric C. Petrie, Gail Li, Earl A. Zimmerman, Dzintra Celmins, Alice D. Brown, Godfrey D. Pearlson, Karen Blank, Karen Anderson, Robert B. Santulli, Tamar J. Kitzmiller, Eben S. Schwartz, Kaycee M. Sink, Jef D. Williamson, Pradeep Garg, Franklin Watkins, Brian R. Ott, Henry Querfurth, Geofrey Tremont, Stephen Salloway, Paul Malloy, Stephen Correia, Jacobo Mintzer, Kenneth Spicer, David Bachman, Dino Massoglia, Nunzio Pomara, Raymundo Hernando, Antero Sarrael, Susan K. Schultz, Laura L. Boles Ponto, Hyungsub Shim, Karen Elizabeth Smith, Amanda Smith, Kristin Fargher, Balebail Ashok Raj, Karl Friedl, Jerome A. Yesavage, Joy L. Taylor, Ansgar J. Furst

**Affiliations:** 1 Biomathematics Laboratory, Department of Applied Mathematics, School of Mathematical Science, Tarbiat Modares University, Tehran, Iran; 2 Applied Systems Biology, Leibniz-Institute for Natural Product Research and Infection Biology – Hans-Knöll-Institute, Jena, Germany; 3 Department of Physiology, Faculty of Medical Sciences, Tarbiat Modares University, Tehran, Iran; 4 UC San Francisco, California, USA; 5 UC San Diego, San Diego, CA USA; 6 Mayo Clinic, Rochester, NY USA; 7 UC Berkeley, Berkeley, CA USA; 8 UPenn, Philadelphia, PA USA; 9 USC, Los Angeles, CA USA; 10 UC Davis, Davis, CA USA; 11 Brigham and Women’s Hospital/Harvard Medical School, Boston, MA USA; 12 Indiana University, Bloomington, IN USA; 13 Washington University St Louis, St Louis, MO USA; 14 Prevent Alzheimer’s Disease, 2131, Rockville, MD USA; 15 Siemens, Munich, Germany; 16 University of Pittsburg, Pittsburg, PA USA; 17 Cornell University, Ithaca, NY USA; 18 Albert Einstein College of Medicine of Yeshiva University, Bronx, NY USA; 19 AD Drug Discovery Foundation, New York, NY USA; 20 Acumen Pharmaceuticals, Livermore, CA USA; 21 Northwestern University, Evanston and Chicago, IL USA; 22 National Institute of Mental Health, Rockville, MD USA; 23 Brown University, Providence, RI USA; 24 Eli Lilly, Indianapolis, IN USA; 25 University of Washington, Seattle, WA USA; 26 University of London, London, England, UK; 27 UCLA, Los Angeles, CA USA; 28 University of Michigan, Ann Arbor, MI USA; 29 University of Utah, Salt Lake, UT USA; 30 Banner Alzheimer’s Institute, Phoenix, AZ USA; 31 UC Irvine, Irvine, CA USA; 32 National Institute on Aging, Bethesda, MD USA; 33 Johns Hopkins University, Baltimore, MD USA; 34 Richard Frank Consulting, Washington, DC USA; 35 Oregon Health and Science University, Portland, OR USA; 36 Baylor College of Medicine, Houston, TX USA; 37 University of Alabama, Birmingham, AL USA; 38 Mount Sinai School of Medicine, New York, NY USA; 39 Rush University Medical Center, Chicago, IL USA; 40 Wien Center, Miami, FL USA; 41 New York University, New York, NY USA; 42 Duke University Medical Center, Durham, NC USA; 43 University of Kentucky, Lexington, KY USA; 44 University of Rochester Medical Center, Rochester, NY USA; 45 University of Texas Southwestern Medical School, Dallas, TX USA; 46 Emory University, Atlanta, GA USA; 47 University of Kansas, Medical Center, Kansas City, KS USA; 48 Mayo Clinic, Jacksonville, FL USA; 49 Yale University School of Medicine, New Haven, CT USA; 50 McGill University/Montreal-Jewish General Hospital, Montreal, QC Canada; 51 Sunnybrook Health Sciences, Toronto, ON Canada; 52 U.B.C. Clinic for AD and Related Disorders, Vancouver, BC Canada; 53 Cognitive Neurology-St Joseph’s, London, ON Canada; 54 Cleveland Clinic Lou Ruvo Center for Brain Health, Las Vegas, NV USA; 55 St Joseph’s Health Care, London, ON Canada; 56 Premiere Research Institute, Palm Beach Neurology, Miami, FL USA; 57 Georgetown University Medical Center, Washington, DC USA; 58 Banner Sun Health Research Institute, Sun City, AZ USA; 59 Boston University, Boston, MA USA; 60 Howard University, Washington, DC USA; 61 Case Western Reserve University, Cleveland, OH USA; 62 Neurological Care of CNY, Liverpool, NY USA; 63 Parkwood Hospital, London, ON Canada; 64 University of Wisconsin, Madison, WI USA; 65 Dent Neurologic Institute, Amherst, NY USA; 66 Ohio State University, Columbus, OH USA; 67 Albany Medical College, Albany, NY USA; 68 Hartford Hospital, Olin Neuropsychiatry Research Center, Hartford, CT USA; 69 Dartmouth-Hitchcock Medical Center, Lebanon, NH USA; 70 Wake Forest University Health Sciences, Winston-Salem, NC USA; 71 Rhode Island Hospital, Providence, RI USA; 72 Butler Hospital, Providence, RI USA; 73 Medical University South Carolina, Charleston, SC USA; 74 Nathan Kline Institute, Orangeburg, NY USA; 75 University of Iowa College of Medicine, Iowa City, IA USA; 76 University of South Florida: USF Health Byrd Alzheimer’s Institute, Tampa, FL USA; 77 Department of Defense, Arlington, VA USA; 78 Stanford University, Stanford, CA USA

**Keywords:** Network topology, Network models, Alzheimer's disease

## Abstract

Alzheimer’s disease (AD) is a progressive disorder associated with cognitive dysfunction that alters the brain’s functional connectivity. Assessing these alterations has become a topic of increasing interest. However, a few studies have examined different stages of AD from a complex network perspective that cover different topological scales. This study used resting state fMRI data to analyze the trend of functional connectivity alterations from a cognitively normal (CN) state through early and late mild cognitive impairment (EMCI and LMCI) and to Alzheimer’s disease. The analyses had been done at the local (hubs and activated links and areas), meso (clustering, assortativity, and rich-club), and global (small-world, small-worldness, and efficiency) topological scales. The results showed that the trends of changes in the topological architecture of the functional brain network were not entirely proportional to the AD progression. There were network characteristics that have changed non-linearly regarding the disease progression, especially at the earliest stage of the disease, i.e., EMCI. Further, it has been indicated that the diseased groups engaged somatomotor, frontoparietal, and default mode modules compared to the CN group. The diseased groups also shifted the functional network towards more random architecture. In the end, the methods introduced in this paper enable us to gain an extensive understanding of the pathological changes of the AD process.

## Introduction

Alzheimer’s disease is an irreversible and progressive brain disorder that is the fifth cause of death worldwide and does not have a pharmacological treatment for cure or prevention^1,2^. AD progression can be considered as a continuum from CN through EMCI and LMCI, and to AD, characterized by the loss of memory and cognitive dysfunction. In more detail, it has been shown that the transition probabilities from MCI to more severe states of AD at age 65 is 14% higher than the transition from CN, and this likelihood is increasing by age^3^. Moreover, the studies show that these disorders are associated with dysfunction in the whole brain neural connectivity rather than a local brain region^4–6^. Therefore, a non-invasive and computational method for modeling the connectivity across the whole brain can help to understand the alterations in the brain network architecture corresponding to the disease progression and provide an opportunity for early diagnosis and understanding of the disease process. In this regard, the use of graph-theoretical methods and complex network theory for modeling the brain as an interconnected network of brain regions have been a focus of interest in recent years^7,8^. In most of the studies, the general framework for developing the brain networks include the selection of spatial and temporal scale and resolution of the study data^9–13^, the appropriate atlases that divide the brain into distinct regions^14^, the type of connection among regions (anatomical, functional, or effective connectivity)^15,16^, and finally the methods of estimating these connectivities^17–19^.

Resting-state functional magnetic resonance imaging (rs-fMRI), which measures the neural activity in the resting state based on the blood-oxygen-level-dependent (BOLD) contrast, has been widely used to estimate the functional connectivity network of the brain^20,21^. Studies indicate the emergence of several complex network phenomena such as assortativity^22,23^, rich-club^24,25^, clustering^26,27^, and small-world^28,29^ in these networks. The modularity and the importance of hub regions have also been discussed in several studies^30–32^. Further, considering the sensitivity of these networks to brain disorders^33–35^, many studies have aimed to develop computational methods for diagnosing and understanding of the disease process^36–38^. Specifically, in the case of AD and its early stages, many studies reported the disturbance of the functional network architecture, indicating the disappearance of clustering^39^ and rich-club^40^ phenomena, alterations to the small-world phenomena^41,42^, decrease in the functional connectivity link weights^43,44^, and the changes in the spatial distribution of the hub regions (high ranked regions) towards the frontal areas of the brain^43,45–47^. However, to the best of our knowledge, none of the studies have done a comprehensive comparative assessment of the functional brain network of patients with AD and its early stages that include all of those subjects mentioned above.

The human brain has an intrinsic multi-scale architecture^48^, which is emerged in different aspects of the data: spatial, temporal, and topological aspects^13^. In this study, the alterations in the human brain functional connectivity network corresponded to the progression of the disease from CN through EMCI and LMCI, and to AD were assessed using multi-scale topological analysis. For each study group, a functional connectivity network of 360 brain regions was computed. The results of different analyses that have been done on these networks are divided into three topological scales: analyses at the scale of the whole network (global), analyses at the single vertex scale (local), and analyses that are in between local and global scales (mesoscale)^13^.

The core question throughout this paper is whether these alterations are following a trend proportional to the disease progression in the continuum from CN to AD. The presence of such trends is perceptible in most of the results in this study. However, in some of them, the trends are not entirely proportional to the disease progression and behave differently in the early stages of the AD spectrum. Further, the analysis showed that the functional brain network tends to shift toward a more randomized architecture by the disease progression. Also, it has been observed that the frontoparietal, somatomotor, and default mode modules are affected more than other modules introduced in the next section.

## Results

The results of this study are mainly obtained from the comparative analysis of 4 group networks, each representing a study group’s functional connectivity network. In order to construct these networks, the fMRI and T1-weighted (T1w) images of 92 subjects (23 subjects for each study group) were passed through a processing pipeline that includes the preprocessing using *fMRIPrep* 20.0.0 (^49,50^, RRID:SCR_016216), extracting 36 nuisance regressors, parcellating the brain surface area into 360 regions, constructing connectivity network among these regions with Pearson correlation coefficient (PCC), computing the mean of the networks that belong to subjects within each study group, and finally, thresholding these four mean networks. The overall processing pipeline can be found in Supplementary Fig. S1. We used an alpha level of .05 for all statistical tests presented in this study.

### Global network analysis

**Figure 1:**
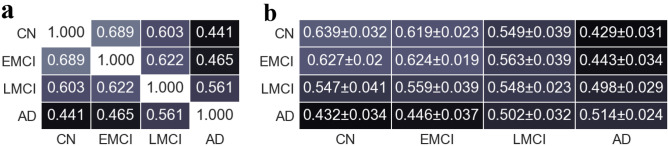
The similarity between study groups follows a trend proportional to the trend of disease progression. (**a**) The Jaccard similarity coefficient between the four main group networks. (**b**) For each study group, the 23 subjects of each group were randomly divided into two subgroups of 11 and 12 networks. Then, the mean networks of these 12 and 11 subjects were constructed, and the Jaccard similarity coefficient between these two mean networks was computed. This process was repeated 100 times resulting in 100 Jaccard similarity coefficient matrices. This panel shows the element-wise mean and SD of these matrices.

Significant differences were revealed at the global level analysis. The Jaccard similarity coefficient is a measure for evaluating the correlation and the similarity among two vectors. This measure which is between 0 and 1, quantifies the similarity among each pair of vectors so that the higher outputs indicate the higher similarity among the elements of these vectors. Computing this measure among vectorized adjacency matrices (a matrix that use to represent a network, where the value of the element (*i*, *j*) of this matrix equals the weight of the link between the vertices *i* and *j*.) corresponded to the 4 group networks were showed that the similarity between the CN network and disease networks were decreased by the progression of the disease (Fig. 1a). However, the existence of similarity between the brain networks of the subjects belonging to the same study group was still unclear. To obtain a more reliable comparison that takes into account these similarities, the samples of each study group were randomly divided into two subgroups of 12 and 11 samples. Similar to the process explained in the “Methods and materials” section for computing the mean network of each study group based on its 23 samples, two different mean networks were generated for each study group based on these two subgroups of samples. Then, the Jaccard similarity coefficient among the 11-samples mean networks and the 12-samples mean networks were computed. This process was repeated 100 times, resulting in 100 similarity matrices . Then, the element-wise mean and standard deviation (SD) over these matrices were computed, and the results showed the existence of almost similar trends (Fig. 1b). Further, this similarity has also been evaluated based on the PCC method and resulted in almost similar trends, which are shown in Supplementary Fig. S2 online.

The mean and SD of vertex strength (the total weight of the vertex connections) also had a decreasing trend. Furthermore, the links were classified into short and long links based on their euclidean length (the short (long) links were defined as the links that the euclidean distance between its vertices is smaller (larger) than the average distance among all vertices.). For each group network, the average weight of the long links and the average weight of the short links was computed. As shown in Table 1, there was a decreasing trend of the long link weights and an increasing trend of the short link weights proportional to the progression of the disease. In general, there was a significant difference among nodal values of group networks shown in Table 1 (*p* < 0.001), indicating that the total strength and the strength of the long links and the intra-module links are decreasing while the strength of the short links, and the inter-module links are increasing by the disease progression.

**Figure 2:**
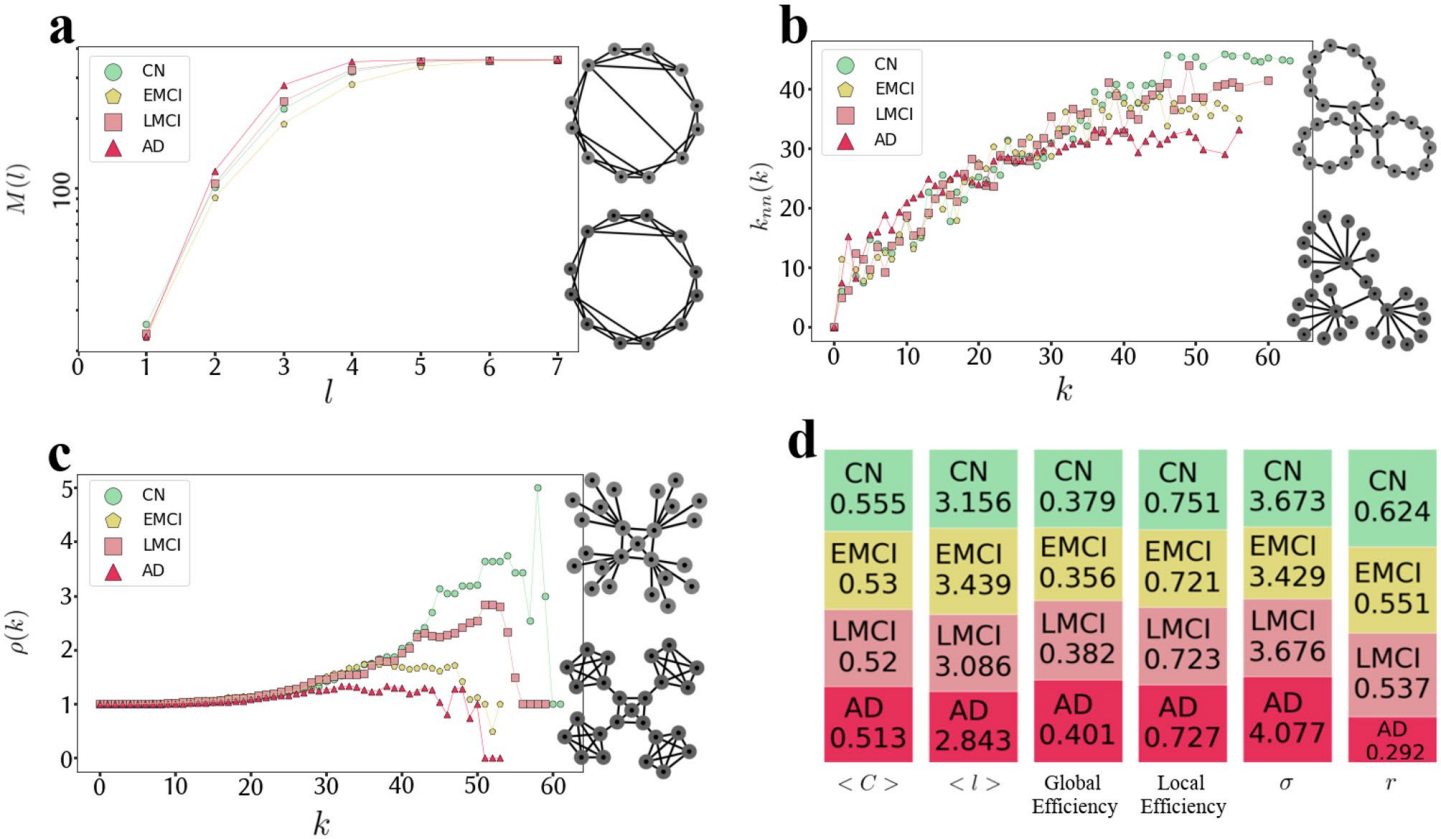
The emergence of the small-worldness, assortativity, and rich-club phenomenon in the group networks. (**a**) *M*(*l*) plot showing the average number of vertices within a distance less than or equal to *l* from any given vertex is almost increased by the disease progression and suggests that the group networks have the small-world property that is almost stronger in the disease networks. Two graphs on the right-hand side are toy examples to provide intuition on the relationship between the small-world property and randomness, demonstrating the increase in the randomness of network connections (from bottom to top), which leads to the increase in the emergence of the small-world property. The bottom network is more regular, whereas the top one is more random. As a result, the average shortest path length is shorter in the random network (top) than the regular one (bottom), causing the regular network to be less small-world. (**b**) $K_{nn}(k)$ plot showing the average degree of the nearest neighbors, for vertices of degree *k*, is almost decreased by the disease progression and suggests that the group networks have assortative architecture, and this assortative matching follows a decreasing trend proportional to the disease progression. Two graphs on the right-hand side are toy examples of assortative (top) and disassortative (bottom) networks to provide intuition on the assortative patterns. As it is obvious, in the bottom network, high degree vertices are more connected with low degree vertices (disassortative pattern), whereas, in the top network, its vertices tend to make connections with other vertices that have similar degrees (assortative pattern). (**c**) $\rho (k)$ plot showing the amount of inter-connectivity among vertices of degree higher than *k* is almost decreased by the disease progression and suggests that the rich-club phenomenon is disappearing by the disease progression. Two graphs on the right-hand side are toy examples of networks with (top) and without (bottom) rich-club. In the bottom network, there is no significant inter-connectivity among high-degree vertices (rich vertices). However, in the top network, which is an example of a rich-club network, high-degree vertices are completely inter-connected. (**d**) The bar plots showing $<C>$, $<l>$, global efficiency, local efficiency, $\sigma$ (normalized $<C>$/ normalized $<l>$), and *r* (PCC between the degrees of all vertices at either ends of a link).

There was also an inverse relationship between the local and global efficiency, which are measures for the efficiency of information exchange over the networks. The global efficiency measures the information exchange over the whole network, whereas the local efficiency measures the fault-tolerant of the network by quantifying the information exchange between the neighbors of each vertex when that vertex is removed^51^. While local efficiency in the CN group was higher than in other groups, global efficiency in the CN group was lower than LMCI and AD groups (Fig. 2d).

The small-world (six degrees of separation) phenomenon describes that it is possible to reach any network vertex from other vertices by passing through a small chain of connected vertices. It has been shown that in small-world networks, the average shortest path length ($<l>$) grows logarithmically with the network size. This phenomenon can be evaluated by measuring $<l>$ and the growth rate of the average number of vertices within a distance less than or equal to *l* from any given vertex (*M*(*l*)). It has been shown that in small-world networks, *M*(*l*) is increasing faster than the exponential growth^52^. The *M*(*l*) function plotted in Fig. 2a and the values of $<l>$, shown in Fig. 2b, both suggest that the group networks have the small-world property. However, these figures indicate that, similar to the global efficiency shown in Fig. 2d; the changing patterns are not quite proportional to the disease progression. Specifically, in almost all of the figures mentioned above, the only study group which is not following the expected trend is the EMCI group. The existence of the small-world phenomenon along with the high value of average clustering coefficient ($<C>$), will lead to a more specific emergence property of complex networks called small-worldness. $\sigma$ which is equals to the division of the normalized $<l>$ by the normalized $<C>$, is a measure of evaluating this new phenomenon explained in the section “Methods and materials”. Figure 2d indicates that in all networks, $\sigma >1$, which means that they all have the small-worldness property. This figure also indicates that $\sigma$, and consequently the small-worldness, increase by the disease progression with the exception of the EMCI group, which is not following the trend. There was also a significant difference between the nodal clustering coefficient (*p* < 0.001) and the shortest path length (p < 0.001) of the group networks, indicating that the networks tend to become less clustered and more small-world by the disease progression.

**Table 1:** General information about the distribution of the links of the study networks.

Study group	CN	EMCI	LMCI	AD	*p*	*F*
**The average strength of the links**						
Total strength	$9.35 \pm 5.4$	$7.84 \pm 4.9$	$7.77 \pm 4.5$	$7.56 \pm 3.8$	< 0.001	8.76
Long links	$0.0068 \pm 0.0494$	$0.0050 \pm 0.0443$	$0.0031 \pm 0.0339$	$0.0029 \pm 0.0323$	< 0.001	60.76
Short links	$0.0500 \pm 0.1360$	$0.0519 \pm 0.1488$	$0.0539 \pm 0.1477$	$0.0541 \pm 0.1506$	< 0.001	46.19
Inter-module links	$0.0022 \pm 0.1031$	$0.0059 \pm 0.0876$	$0.0092 \pm 0.0861$	$0.0108 \pm 0.1018$	< 0.001	146.3
Intra-module links	$0.1695 \pm 0.1478$	$0.1493 \pm 0.1274$	$0.1309 \pm 0.1178$	$0.1218 \pm 0.1314$	< 0.001	28.13

Pairwise comparison	CN-AD	EMCI-AD	LMCI-AD	CN-EMCI	CN-LMCI	EMCI-LMCI
**The pairwise t-test comparison**						
Total strength	< 0.001	0.287	0.002	0.001	0.234	0.038
Long links	< 0.001	< 0.001	0.001	0.061	< 0.001	< 0.001
Short links	< 0.001	0.911	< 0.001	< 0.001	< 0.001	< 0.001
Inter-module links	< 0.001	< 0.001	< 0.001	< 0.001	< 0.001	0.054
Intra-module links	0.190	< 0.001	0.200	< 0.001	0.010	< 0.001

The top table shows the average strength of the links, which are grouped into long or short links and inter-module or intra-module links. The significance of the pairwise differences is assessed using the t-test and is shown in the bottom table.

### Mesoscale network analysis

The analysis that had been done in the mesoscale indicates the existence of trends similar to the analyses at the global scale.

#### Clustering, assortativity, and rich-club

The clustering, assortativity, and rich-club phenomena have been used to describe the higher-level architecture of a complex network. The high value of $<C>$ indicates the presence of highly interconnected groups of vertices within the network. This measure which is depicted in the second bar plot of Fig. 2d, shows that the clustering phenomenon is decreasing with the disease progression. The assortativity phenomenon is characterized when neighbor vertices are likely to have similar degrees. In Fig. 2b, the increasing diagram of the average degree of the nearest neighbors for vertices of degree *k* ($k_{nn}(k)$) indicates the presence of this phenomenon. This figure and the last bar plot in Fig. 2d showing the PCC between the degrees of all vertices at either ends of a link (*r*) in each group network, also indicate that the assortativity is disappearing by the disease progression. The rich-club phenomenon is characterized when large degree vertices are more interconnected with each other than with the smaller degree vertices. The diagram in Fig. 2c plotting the fraction of links connecting vertices with degree higher than *k* out of the maximum number of links that these vertices can possibly share ($\rho (k)$), indicates the presence of the rich-club phenomenon, which is disappearing by the disease progression.

#### Modular analysis

**Figure 3:**
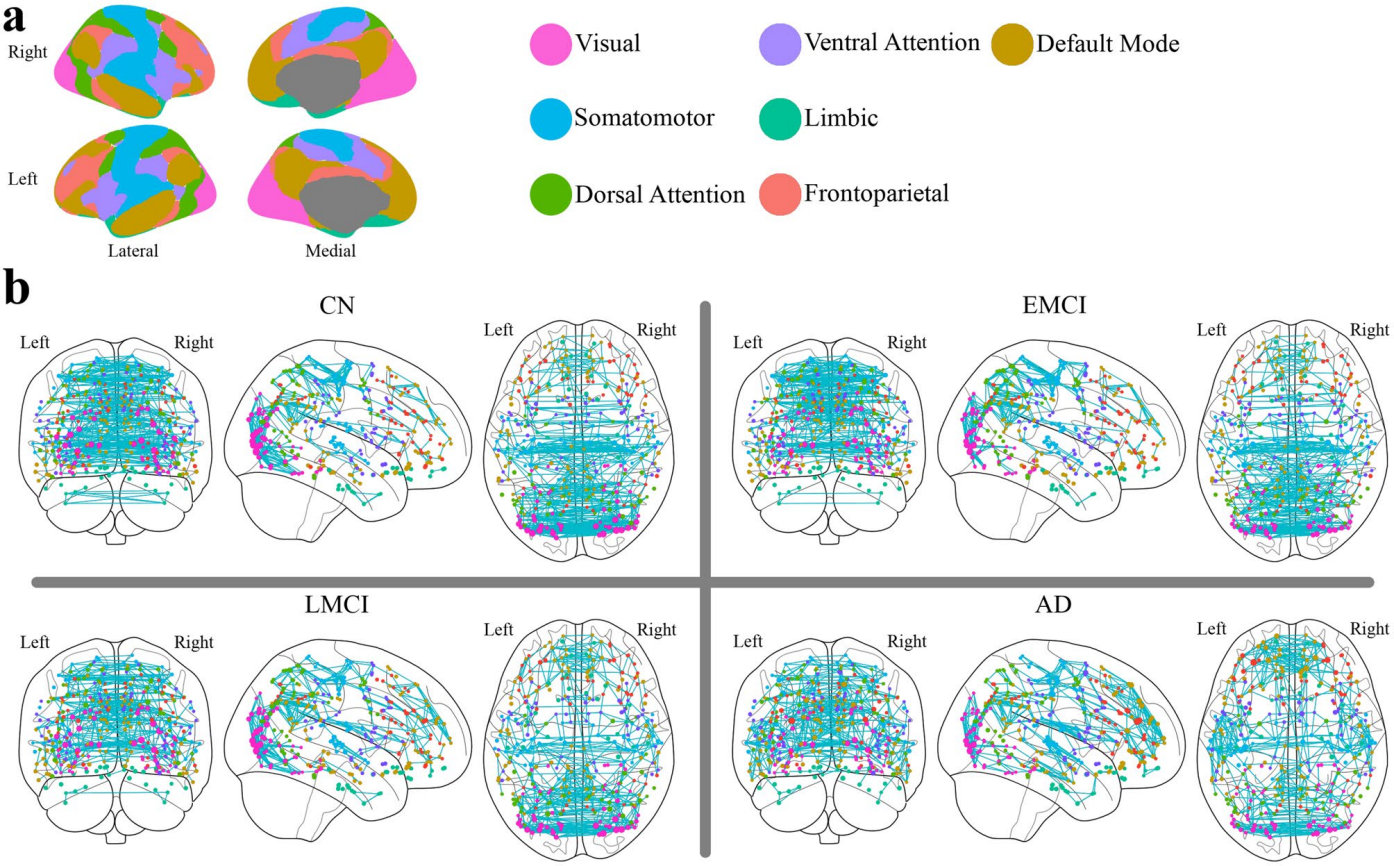
The overall gradually changing patterns of highly-weighted links are following a trend proportional to the disease progression. (**a**) The spatial distribution of the 7-module parcellation. (**b**) The top 1 percent highly-weighted links of each group network. Vertex color represents the module the vertex belongs to, and the vertex size is proportional to the vertex strength. It shows that there is a trend towards the increase in the number of default mode links as well as the decrease in the inter-hemisphere links in dorsal attention. Visualizations were created using *ggseg* 1.6.1 R package^54^ (**a**) and *Nilearn* 0.6.2 (https://nilearn.github.io) (**b**).

The 360 brain regions were classified into seven modules based on a well-known parcellation provided by Yeo et al.^53^ (Fig. 3a). Then, the topological architecture of intra-module and inter-module connection networks was analyzed. First, by reorganizing the position of vertices in each group network’s adjacency matrix based on this classification, a substantial structure that was disappearing with the progression of the disease was observed (see Supplementary Fig. S3 online). Then, the average weight of the intra-module links and the average weight of the inter-module links were computed for each group network. As it is shown in Table 1, there was an increasing trend of the inter-module link weights and an increasing trend of the intra-module link weights proportional to the progression of the disease, and there was a significant difference among nodal values of group networks (*p* < 0.001). Then, similar to the “Global network analysis” section where the PCC between adjacency matrices of the group networks was measured, for each module, the PCC was computed among the adjacency matrices of the modular sub-network of each group network. The results showed that these modules are sensitive to the disease progression (see Supplementary Fig. S4 online).

### Local network analysis

Several approaches have been attempted to attain a general conclusion about the distribution of the regions and links that are most sensitive to the disease progression at the finest topological scale.

**Figure 4:**
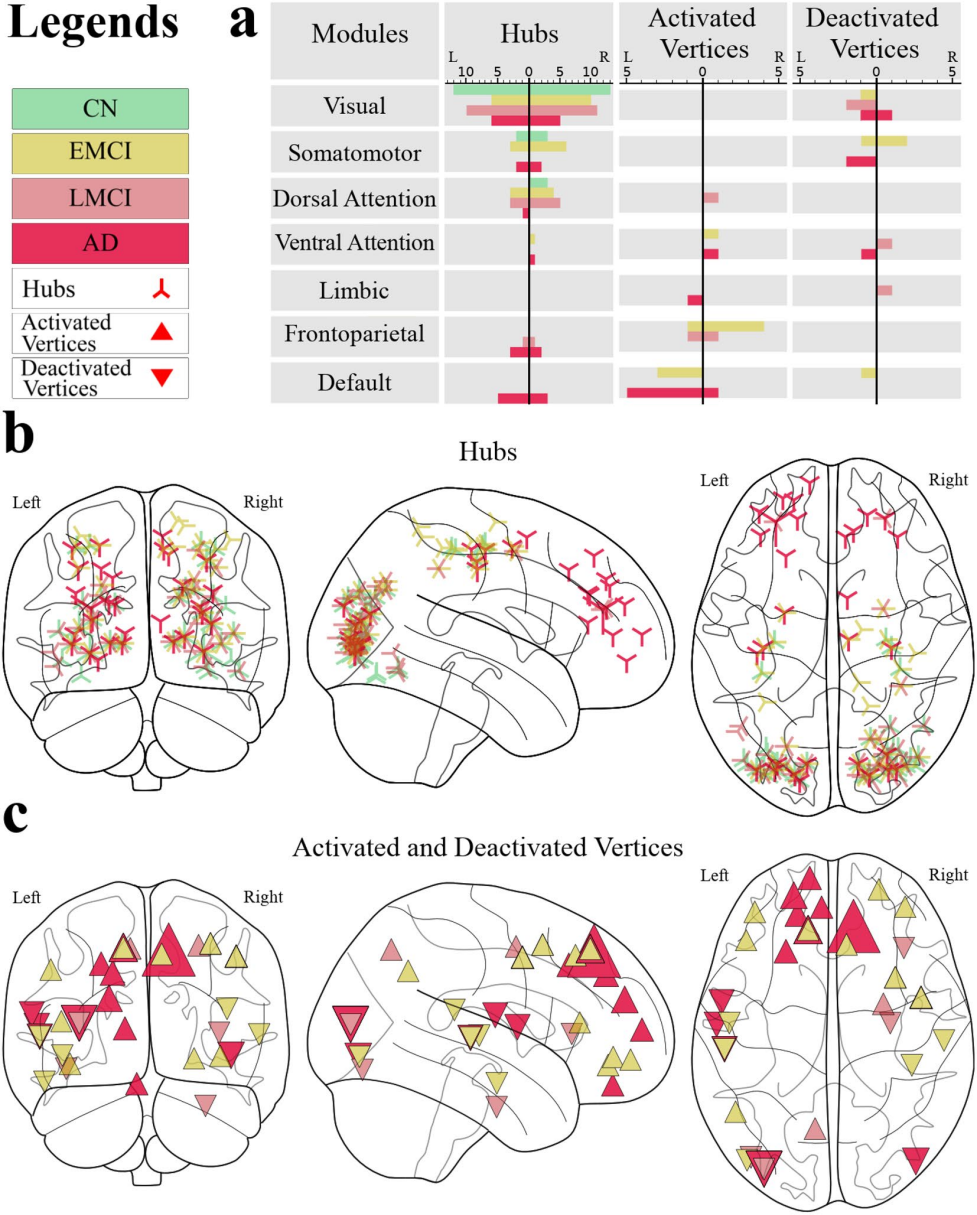
The distribution of hubs, activated areas, and deactivated areas shows that anterior regions are more engaged in the diseased groups. (**a**) The frequency distribution across the 7-module parcellation. The left and right sides of each sub-plot denoted by a vertical black line represent the distribution across modules limited to the left and right hemispheres, respectively (**b**) The spatial distribution of the hub regions. The vertex size is proportional to the vertex strength. (**c**) The spatial distribution of vertices that are activated (denoted by $\bigtriangleup$) and deactivated (denoted by $\bigtriangledown$) in the diseases. A vertex was called activated (deactivated) with respect to disease if the subtraction of its strength in the disease network from the CN network was significantly larger (smaller) than the similar value for other vertices. The vertex size is proportional to the value of this subtraction. Visualizations in (**b**) and (**c**) were created using *Nilearn* package.

In order to attain the most sensitive vertices, the spatial distribution of the hub vertices (vertices whose strength is higher than $mean + 2\times SD$ of all vertices) and the diseased-related activated and deactivated vertices were calculated (Fig. 4b,c). Further, the frequency distribution of these vertices across the 7-module parcellation introduced in the “Modular analysis” section is shown in Fig. 4a.

**Figure 5:**
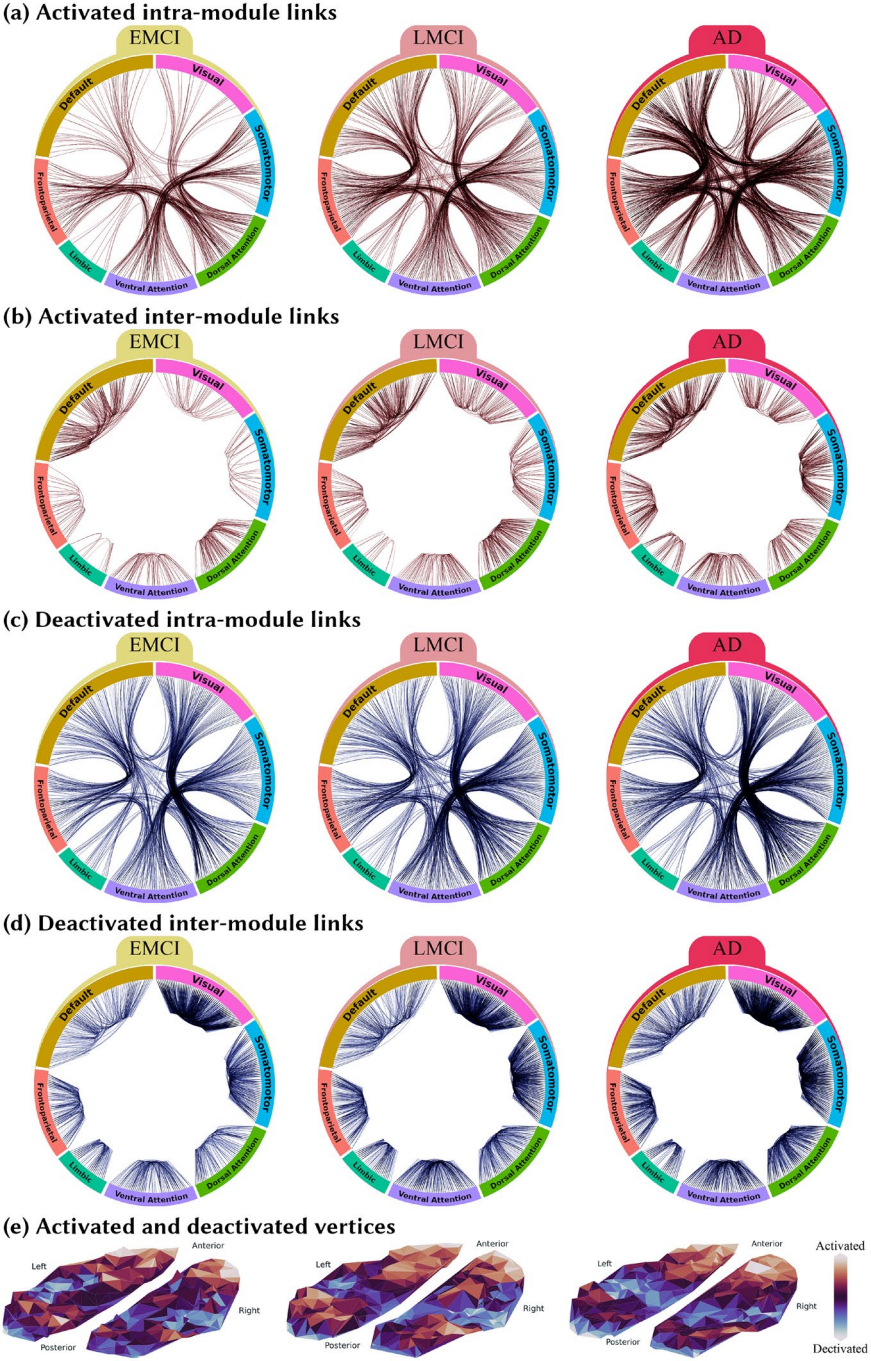
The alterations in the brain functional network induced by the disease progression. For each disease stage, the connectogram of the links with significantly higher (activated) or lower (deactivated) weights compared to the CN group is depicted in the top four panels: (**a**) connectogram of intra-module links activated in each disease stage compared to the CN group, (**b**) Connectogram of inter-module links activated in each disease stage compared to the CN group, (**c**) connectogram of intra-module links deactivated in each disease stage compared to the CN group, and (**d**) Connectogram of inter-module links deactivated in each disease stage compared to the CN group. For each disease group, the activated (deactivated) links compared to the CN group are defined as the significantly larger (smaller) elements of the matrix resulting from the subtraction of the disease network from the CN network. Diagrams are plotted based on the *Hierarchical Edge Bundling* algorithm^55^ using *ObservableHQ* platform (https://observablehq.com). This algorithm allows bundling the intra-module links to get a more elegant presentation. Panel (**e**) indicates the activation or deactivation in the vertices’ strength by showing the top-view spatial distribution of the activated or deactivated vertices similar to Fig. 4c.

In order to attain the most sensitive links, for each disease stage two networks including the significantly activated and deactivated links in the diseased network with respect to the CN network were constructed. As it is obvious in Fig. 5a–d, These networks show a similar connectivity pattern among the seven modules, which is becoming more and more apparent with disease progression. Further, we have statistically evaluated the significance of the patterns in the disease-related activated or deactivated networks, presented in Fig. 5. In order to do that, we first categorized the links based on whether they were inter-module or intra-module. Then we used the ANOVA test to check if there is a significant difference between the strength of the links belonging to different inter-module (intra-module) categories. The results showed that these differences were significant with *p* < 0.001 for all disease groups.

Also, the overall gradually changing pattern of the top 1 percent highly-weighted links of the group networks is depicted in Fig. 3b. It shows a changing pattern toward increasing the number of default mode links and decreasing the inter-hemisphere links in the dorsal attention module. Further, the significance of these trends were statistically evaluated using the Chi-squared test with *p* < 0.001 for default mode links and *p* = .005 for intra-hemisphere links in dorsal attention. In order to do that, we computed the number of inter-module (intra-module) links that appeared among the top 1 percent strongest links of each group network and also the number of inter-module (intra-module) links that did not appear among them. Then we used the chi-squared test to see whether these numbers were significantly different across study groups. We further categorized the links based on both their module and the hemisphere they belong to and repeated the similar chi-squared test. In Supplementary information, Fig. S5, we have provided detailed axial images indicating the brain areas in which the trends have appeared. Also, a larger figure similar to Fig. 3b has been added to the Supplementary information (Fig. S6).

## Discussion

The main goal of this study was to investigate the alterations of the brain functional network topology in patients with AD, LMCI, and EMCI compared to the CN patients from a multi-scale topological perspective. This study attempted to find quantitative network measures that are proportional to the trend of brain deterioration from CN patients through AD in order to suggest network-based biomarkers and also help to understand the disease process. There are three general findings in this study. First, the functional brain network constructed in this study is sensitive to the progression of AD. Second, the functional brain network of the diseased groups tends to shift towards a more randomized and integrated architecture. Third, the present study suggests that the alterations in the network architecture may not be entirely proportional to the disease progression. In this regard, the functional brain network architecture may go through a nonlinear process and behave differently in earlier stages in the course of the disease progression.

Several study results suggested that the networks of the disease groups have larger global efficiency. These results include the increase in the global efficiency, the small-world property, and the proportion of inter-module to intra-module link weights. There are also several results suggesting the smaller local efficiency. These results include the decrease in the local efficiency, $<C>$, and the proportion of intra-module to inter-module link weights. This inverse relationship which is reported by previous brain studies^56,57^ can be explained by considering the increase of global efficiency as a compensatory mechanism for the decrease in the local efficiency. This will leads to the efficiency in information spreading across the whole brain but the loss of efficacy in information spreading in the finer scales of vertices and their neighbors. Considering the sparse nature of the brain networks, the increase in the global and decrease in the local efficiency indicate that the disease networks have a more random organization.

The analysis at the mesoscale revealed the alteration in the brain network organization in a way that causes the disappearance of the assortative mixing and the rich-club phenomenon. $\rho (k)$ is declining by the disease progression, and in the case of the AD network, it is around 1 in almost everywhere, which means that the brain network tends to behave more similarly to random networks. This significant rich-club reorganization magnifies the importance of analyzing the alterations of the hub regions. The diseased networks’ hubs are more distributed over the modules, and it is consistent with the claim that the diseased networks are more randomized and integrated. Furthermore, the diseased groups have significantly more hubs located in anterior regions as compared to the CN group. In more detail, the analysis of activated and deactivated areas shows that the default mode, frontoparietal, and somatomotor modules have been significantly affected by the disease. As suggested by Sha et al.^58^, this observation can find its explanation in the functionality of these modules: frontoparietal is responsible for cognitive control, the default mode network is involved in internal emotional processing, self-referential directed thought, and memory function^58^, and somatomotor is responsible for motor skill learning and sensory perception. Furthermore, As it is also reported by other studies^59,60^, in addition to these modules, the dorsal and ventral attention, which are respectively involved in the top-down and bottom-up attention process, are also significantly involved in the networks of the disease-related alterations.

As described in the “Network efficiency” subsection, the quality of being random or regular in complex networks is closely related to global and local efficiency, small-worldness, average clustering, and average shortest path length. Further, in the AD network, the disappearance of rich-club organization, the location hub regions more uniformly distributed over the seven modules, and the decrease in the values of Fig. 1b indicates the increase in the AD network randomness. In general, the present analysis results suggest that the brain network tends to shift towards a random structure by the disease progression. There are also several measures for assessing the integration in brain networks, including the decrease in average clustering and the intra-module link weights, as well as the increase in average shortest path length and the inter-module link weights.

Despite several advantages in this study, some limitations should be addressed. First, due to the ill-posed nature of constructing functional network problems and due to the lack of ground truth in any of the main steps for generating functional networks out of neuroimaging data, many different strategies can be used for this purpose^61^. Previous studies have shown that depend on the type of analysis, different strategies can lead to different results^14,62^. Therefore, a comparative analysis of different strategies would help choose appropriate strategies depending on the different scales of topological network analysis and investigate whether a single strategy would be appropriate for all scales. Second, Because of the same reasons mentioned in the first limitation, it can be difficult to distinguish which strategy leads to the results that present a more valid biological interpretation^61^. In the case of using these results for developing machine learning models, this lack of interpretability is acceptable, but interpreting these results in order to understand the disease process and the underlying mechanisms that lead to the disease, should be done more carefully.

In conclusion, the analyses offered in this paper can help to gain an understanding of the topological changes in the functional brain network architecture. Most previous studies for analyzing the AD spectrum, focused on one topological scale. Using a multi-scale topological approach, this paper extensively demonstrated the pathological changes in the AD process. The issues mentioned in the limitations are the subjects of future works. A comparative assessment of different functional networks constructed based on widely used strategies will help to find strategies with higher sensitivity to the AD spectrum.

## Methods and materials

### Demographic and clinical information

Data used in the preparation of this article were obtained from the ADNI database (adni.loni.usc.edu). Hundreds of patients took part in the ADNI project, which launched in 2003 as a public-private partnership, led by Principal Investigator Michael W. Weiner, MD. The primary goal of ADNI has been to test whether serial magnetic resonance imaging (MRI), positron emission tomography (PET), other biological markers, and clinical and neuropsychological assessment can be combined to measure the progression of MCI and early AD. All statements were performed in accordance with relevant guidelines and regulations. Table 2 shows the demographic and clinical information of the 92 study subjects, which are selected from a sample of 96 subjects from the ADNI2 dataset. After estimating a correlation matrix for each participant, in each disease group, a participant with less similarity among the group participants was excluded. The ANOVA assessment showed that the study groups are aged-matched (F(3,88)=1.00149, p=0.396122.). Also, except for the GDSCALE, other clinical information presented in Table 2, were significantly different across the study groups.

The information provided in Table 2 is obtained from different clinical and psychological tests. The Mini-Mental State Examination (MMSE) is a 30-point questionnaire that is the most common test for measuring cognitive impairment used in clinical assessments^63^. The Geriatric Depression Scale (GDS) is a 30-item yes-or-no questionnaire about the participants’ feelings over the past week^64^. The Global Clinical Dementia Rating (Global CDR) is a rating scale for staging patients diagnosed with dementia^65^. The Functional Activities Questionnaire (FAQ) measures instrumental activities of daily living, such as housework and shopping^66^. The Neuropsychiatric Inventory Questionnaire (NPI-Q) measures the presence of Neuropsychiatric Symptoms^67^.

Many studies have recently viewed the AD continuum as a continuous spectrum characterized by biomarkers and neuropathological findings^68–70^. However, in the case of using functional brain network analysis for studying the progression of AD, subdividing the continuum into distinct categories based on their clinical symptoms has been widely used to represent the AD continuum^71,72^. It is worth mentioning that a growing number of studies use biomarkers based on the levels of Amyloid-beta and p-tau proteins in cerebrospinal fluid^73,74^. The criteria that ADNI2 used to classify Alzheimer’s disease continuum into four stages (CN, EMCI, LMCI, and AD), is mainly based on MMSE score, Wechsler Memory Scale, and global CDR. More details on the inclusion/exclusion criteria are available on the ADNI2 protocol document (adni.loni.usc.edu/wp-content/themes/freshnews-dev-v2/documents/clinical/ADNI-2_Protocol.pdf). The fMRI and T1w images of 23 samples for each study group were collected from the ADNI database of hundreds of patients. According to the ADNI data acquisition protocol, Siemens (Slice Thickness = 4.0; TE = 13.0; TR = 3400.0) and Philips (Slice Thickness = 3.3; TE = 30.0; TR = 3000.0) scanners with Field Strength=3.0 were used for data acquisition. It is worth mentioning that, a growing number of studies recently subdivided the MCI study cohort on the basis of AD-pathological load^75,76^. In this study, the main reason for choosing the four stage categorization is the existence of many articles that use the same categorization for analyzing AD via network science^77–79^, and it provides a better opportunity to compare and validate our results with the other’s findings.

**Table 2:** Demographic and clinical information of the studied groups is shown in the top table.

Group	CN	EMCI	LMCI	AD	*p*	*F*
**Demographic and clinical information**						
Number	23	23	23	23	-	-
Male, female	8,15	12,11	10,13	11,12	.673	-
Age (mean ± SD)	$75.82 \pm 9.44$	$75.29 \pm 6.36$	$72.26 \pm 7.05$	$77.75 \pm 3.75$	.396	1.00
MMSE (mean ± SD)	$28.96 \pm 1.17$	$28.32 \pm 1.89$	$25.87 \pm 3.86$	$20.32 \pm 4.26$	< 0.001	33.16
GDSCALE (mean ± SD)	$1.04 \pm 1.41$	$1.80 \pm 1.85$	$1.94 \pm 2.29$	$1.37 \pm 1.32$	.588	0.65
Global CDR (mean ± SD)	$0.40 \pm 0.13$	$0.39 \pm 0.24$	$0.49 \pm 0.25$	$0.96 \pm 0.40$	< 0.001	46.41
FAQ (mean ± SD)	$0.25 \pm 1.22$	$2.77 \pm 4.22$	$5.06 \pm 6.68$	$18.26 \pm 7.87$	< 0.001	46.61
NPI-Q (mean ± SD)	$0.67 \pm 1.54$	$2.47 \pm 2.52$	$2.08 \pm 1.98$	$4.85 \pm 3.78$	.002	5.41

Pairwise comparison	CN-AD	EMCI-AD	LMCI-AD	CN-EMCI	CN-LMCI	EMCI-LMCI
**The pairwise t-test comparison**						
Age	0.932	0.539	0.202	0.878	0.512	0.919
MMSE	< 0.001	< 0.001	< 0.001	0.880	0.047	0.233
GDSCALE	0.817	0.961	0.982	0.523	0.596	0.999
Global CDR	< 0.001	< 0.001	< 0.001	< 0.001	< 0.001	0.515
FAQ	< 0.001	< 0.001	< 0.001	0.582	0.060	0.581
NPI-Q	0.429	0.633	< 0.001	0.987	0.067	0.029

The pairwise t-test comparisons of the clinical information are presented in the bottom table.

### Preprocessing

The neuroimaging data was preprocessed using *fMRIPrep* 20.0.0, which is based on *Nipype* 1.4.2 (^80,81^, RRID:SCR_002502).

#### Anatomical data preprocessing

The T1w image was corrected for intensity non-uniformity (INU) with ‘N4BiasFieldCorrection’^82^, distributed with ANTs 2.2.0^83^, RRID:SCR_004757], and used as T1w-reference throughout the workflow. The T1w-reference was then skull-stripped with a *Nipype* implementation of the ‘antsBrainExtraction.sh’ workflow (from ANTs), using OASIS30ANTs as target template. Brain tissue segmentation of cerebrospinal fluid (CSF), white-matter (WM) and gray-matter (GM) was performed on the brain-extracted T1w using ‘fast’ [FSL 5.0.9, RRID:SCR_002823^84^]. Brain surfaces were reconstructed using ‘recon-all’ [FreeSurfer 6.0.1, RRID:SCR_001847^85^], and the brain mask estimated previously was refined with a custom variation of the method to reconcile ANTs-derived and FreeSurfer-derived segmentations of the cortical gray-matter of Mindboggle [RRID:SCR_002438^86^]. Volume-based spatial normalization to one standard space (MNI152NLin2009cAsym) was performed through nonlinear registration with ‘antsRegistration’ (ANTs 2.2.0), using brain-extracted versions of both T1w reference and the T1w template. The following template was selected for spatial normalization: *ICBM 152 Nonlinear Asymmetrical template version 2009c*^87^, RRID:SCR_008796; TemplateFlow ID: MNI152NLin2009cAsym].

#### Functional data preprocessing

For each of the BOLD runs found per subject (across all tasks and sessions), the following preprocessing was performed. First, a reference volume and its skull-stripped version were generated using a custom methodology of *fMRIPrep*. Susceptibility distortion correction (SDC) was omitted. The BOLD reference was then co-registered to the T1w reference using ‘bbregister’ (FreeSurfer) which implements boundary-based registration^88^. Co-registration was configured with six degrees of freedom. Head-motion parameters with respect to the BOLD reference (transformation matrices, and six corresponding rotation and translation parameters) are estimated before any spatiotemporal filtering using ‘mcflirt’ [FSL 5.0.9^89^]. The BOLD time-series were resampled onto the following surfaces (FreeSurfer reconstruction nomenclature): *fsaverage5*. The BOLD time-series (including slice-timing correction when applied) were resampled onto their original, native space by applying the transforms to correct for head-motion. These resampled BOLD time-series will be referred to as *preprocessed BOLD in original space*, or just *preprocessed BOLD*. The BOLD time-series were resampled into standard space, generating a *preprocessed BOLD run in MNI152NLin2009cAsym space*. First, a reference volume and its skull-stripped version were generated using a custom methodology of *fMRIPrep*. Several confounding time-series were calculated based on the *preprocessed BOLD*: framewise displacement (FD), DVARS and three region-wise global signals. FD and DVARS are calculated for each functional run, both using their implementations in *Nipype* [following the definitions by^90^]. The three global signals are extracted within the CSF, the WM, and the whole-brain masks. Additionally, a set of physiological regressors were extracted to allow for component-based noise correction [*CompCor*^91^]. Principal components are estimated after high-pass filtering the *preprocessed BOLD* time-series (using a discrete cosine filter with 128s cut-off) for the two *CompCor* variants: temporal (tCompCor) and anatomical (aCompCor). tCompCor components are then calculated from the top 5% variable voxels within a mask covering the subcortical regions. This subcortical mask is obtained by heavily eroding the brain mask, which ensures it does not include cortical GM regions. For aCompCor, components are calculated within the intersection of the aforementioned mask and the union of CSF and WM masks calculated in T1w space, after their projection to the native space of each functional run (using the inverse BOLD-to-T1w transformation). Components are also calculated separately within the WM and CSF masks. For each CompCor decomposition, the *k* components with the largest singular values are retained, such that the retained components’ time series are sufficient to explain 50 percent of variance across the nuisance mask (CSF, WM, combined, or temporal). The remaining components are dropped from consideration. The head-motion estimates calculated in the correction step were also placed within the corresponding confounds file. The confound time series derived from head motion estimates and global signals were expanded with the inclusion of temporal derivatives and quadratic terms for each^92^. Frames that exceeded a threshold of 0.5 mm FD or 1.5 standardised DVARS were annotated as motion outliers. All resamplings can be performed with *a single interpolation step* by composing all the pertinent transformations (i.e. head-motion transform matrices, susceptibility distortion correction when available, and co-registrations to anatomical and output spaces). Gridded (volumetric) resamplings were performed using ‘antsApplyTransforms’ (ANTs), interpolation to minimize the smoothing effects of other kernels^93^. Non-gridded (surface) resamplings were performed using ‘mri_vol2surf’ (FreeSurfer).

Next, confound regression with 36-parameters (including 6 motion parameters of translation and rotation, mean signal in white matter, mean signal in cerebrospinal fluid, and global signal, as well as their derivatives, quadratic terms, and squares of derivatives) were conducted to the data resampled onto FreeSurfer *fsaverage5* surface space^92^.

### Network construction

The human connectome project’s multi-modal parcellation, version 1.0 (HCP_MMP 1.0)^94^ were used to parcellate each participant’s data into 360 regions. It has been shown that this parcellation is more robust and sensitive to AD progression than many other widely used parcellations, and it is probably the most detailed cortical in-vivo parcellation available to date^14,95^. The time series corresponding to each region is then the mean of the time series of voxels within that region. Then, the weighted undirected functional connectivity network was obtained by computing the PCC between time series of all regions. In order to obtain a single network for each study group, the mean network of each group was computed as the element-wise mean of all networks belong to the subjects within that group. Finally, instead of using a constant threshold value to remove weak links in each group, we used a data-driven method to compute a threshold value. This new criterion is inspired by the fact that complex networks have strong global efficiency as well as sparsity. Furthermore, it has been shown that this approach has a good potential for the diagnosis of Alzheimer’s disease. For each network, we searched for a thresholding value that maximizes the global efficiency (*E*) minus the proportion of the strongest weights (*PSW*) (Eqs. 1, 2)^96^.

1 $$\begin{aligned} E(t) = \frac{1}{n}\sum _{i\in N}^{}\frac{\sum _{j\in N,j\ne i}^{ }(\frac{1}{d_{ij}})}{n-1} \end{aligned}$$

2 $$\begin{aligned} PSW(t) = \frac{\sum _{i\in W,i\ge t}^{}i}{\sum _{j\in W}^{}j} \end{aligned}$$

where *N* is the set of all vertices, *n* is the number of vertices, $d_{ij}$ is the shortest path length between vertices *i* and *j*, and *W* is the set of all weights. However, the results of this method were not very different from using the same thresholds for each group, and the resulting thresholds were close to each other (CN: 0.093, EMCI: 0.086, LMCI: 0.101, AD: 0.083).

### Network analysis

The study of large-scale complex networks includes a wide range of statistical measures that have been defined for the study of large-scale complex networks as characteristics for network architectures. Most of these measures have specific real-world interpretations. However, some of them have been shown to be more manifested in real-world networks, and more specifically the brain networks, representing their intrinsic complexity^97,98^ (e.g., small-worldness and efficiency), heterogeneity^99^ (e.g., degree distribution, hubs), hierarchical structure^24,100^ (e.g., rich-club, assortativity, clustering, and hubs), integration (e.g., small-world, clustering, local efficiency), and segregation (e.g., average shortest path length, global efficiency)^32,101^. This study used a collection of most dominant measures that can evaluate these features and also capture different topological scales^13^. Further, these measures have been widely used in studying the AD continuum^40,47,102^.

#### Network efficiency

The efficiency of information exchange throughout the networks can be assessed from local and global perspectives^51,103,104^.

Having a small $<l>$ leads to efficient information exchange from the global point of view. Accordingly, several quantitative methods were introduced to assess this efficiency. The global efficiency, which is the inverse sum of the shortest path length among vertices, is one of them^51^. The small-world property which is a common emergence phenomenon among many real-world complex network systems, also guarantees high efficiency in the information exchange from the global perspective. This measure that also calls the six degrees of separation, exists if $<l>$ scales slower than the logarithmic growth with the size of the network. *M*(*l*) is defined as the average number of vertices within a distance less than or equal to *l* from any given vertex. Then *M*(*l*) is expected to grow faster than the exponential growth in the small-world networks^52^.

It has been shown that many real-world complex networks are mostly sparse networks with high $<C>$^105^ that will lead to the efficiency in the local information exchange. This efficiency can also be assessed by measuring the average efficiency of the sub-networks, including each vertex and its neighbors^51^. Clearly, this efficiency can not be attained if the network has a completely random organization. However, the sparse nature of these networks, along with the existence of the small-world property, implies that these networks can not be like regular networks, and there should be random rearrangements in the connections of these networks to form hubs and connectivity backbones and leads to faster information exchange and decrease in $<l>$^52^. Therefore, the emergence of both local and global efficiency, which can be explained by the trade-off between the regular and random organization of the connections^103^, was recognized as a new phenomenon, called the small-worldness^106^. A straightforward approach for quantifying this phenomenon is to divide the normalized $<l>$ by the normalized $<C>$ ($\sigma =<l>_{rand}/C_{rand}$). The normalization can be done by dividing the actual value of each measure by the value of the measure for a corresponding Erdős-Rényi random graph. A network is then said to have small-worldness if $\sigma >1$^106^.

#### Assortativity

The assortative property refers to the tendency of vertices to connect to other vertices with similar degree. In this study, this property was measured using two methods. First, the PCC between the degrees of all vertices on either ends of a link was computed as:

3 $$\begin{aligned} r = \frac{\sum _{e}^{}j_{e}k_{e}/E-{[\sum _{e}^{}(j_{e}+k_{e})/(2E)]}^2}{[\sum _{e}^{}({j_{e}}^2+{k_{e}}^2)/(2E)]-[{\sum _{e}^{}(j_{e}+k_{e})/(2E)}^2]} \end{aligned}$$

where $j_{e}$ and $k_{e}$ denote the degree of the extremities of link *e* and *E* is the total number of links. This quantity lies in the range $-1\le r\le 1$, where $-1$ refers to disassortative networks and 1 refers to totally assortative networks^107^. The second method measures the assortativity by computing the average degree of the nearest neighbors, for vertices of degree *k* ($k_{nn}(k)$).

4 $$\begin{aligned} k_{nn}(k) = \frac{1}{N_{k}}\sum _{i/k_{i}=k}^{}k_{nn,i} \end{aligned}$$

where $N_{k}$ is the number of vertices with degree *k* and $k_{nn,i}$ is the average degree of the nearest neighbors, for the vertex *i*. If $k_{nn}(k)$ is an increasing function of *k*, the average degree of the nearest neighbors, for vertices of degree *k* will increases by *k* which means that the probability of these vertices being connected with large degree vertices is increasing and this corresponds to an assortative mixing^107^.

#### Rich-club

The rich-club property indicates the tendency of high degree vertices, to be connected to each other and forming clubs. It can be quantitatively measured as:

5 $$\begin{aligned} \phi (k) = \frac{2 E_{>k}}{N_{>k}(N_{>k}-1)} \end{aligned}$$

where $E_{>k}$ is the number of links among the vertices with degree larger than *k*, and $N_{>k}$ is the number of vertices with degree larger than *k*. In order to obtain a more representative measure, the $\phi (k)$ of the network under study was normalized by dividing with the $\phi (k)$ of a random network with the same degree distribution as the network under study. This function is called $\rho _{ran}(k)$. If $\rho _{ran}(k)$ is an increasing function of *k*, the sub-networks containing vertices with degree larger than *k* will be denser as *k* increases and this implies the presence of a rich-club organization^108^.

#### Sensitive vertices and links

Given a scoring system for vertices, network hubs are defined as the vertices whose score is significantly higher than others ($> mean + 2\times SD$). In this study, we used strength as the scoring system. Additionally, by defining a new score as the subtraction of the score of the network under study (in here the disease networks) from a reference network (in here the CN network), the activated regions in that network with respect to the reference network, is then computed as the vertices that their new score is significantly higher than others, respectively the deactivated regions are the ones that their new score is significantly lower. Further, a scoring system for network links was defined by computing the difference between the adjacency matrix of the disease networks and the CN network. Likewise, for each disease group, the activated (deactivated) links were determined as links whose score is significantly higher (lower) than others.

#### Statistical analyses

In General, One-Way ANOVA and Chi-squared ($\chi ^2$) tests have been performed for continuous or categorical variables, respectively, throughout the results. Additionally, Kruskal-Wallis one-way analysis of variance, which is a nonparametric alternative to the One-Way ANOVA test was used in this analysis (all with *p* < 0.05).

Further, As it is also explained in the “Rich-club” and “Network efficiency” subsections, in order to assess the significance of the rich-club and small-worldness, the $\phi (k)$ and the $\sigma$ functions were included a standardization part in which these functions were computed for a collection of 1000 randomly generated networks with the same degree distribution as the original networks (based on edge_swap algorithm).

The main approach of this study was to perform complex network analysis on the brain connectivity in each disease stage. Therefore, one goal was to estimate a single brain network for each stage of the disease. To do so, as many other studies suggest^109–112^, averaging the networks over all participants within each group was the best option for estimating the group networks. Indeed, one concern in this study was whether the group averaged networks could represent the group participants. In this regard, a permutation test was designed that repetitively creates two average networks for each disease group using random permutations and investigates whether these average networks are more similar to each other than the average networks of other diseases (Fig. 1b).

## Supplementary Information

Supplementary Information 1.

Supplementary Information 2.

Supplementary Information 3.

Supplementary Information 4.

Supplementary Information 5.

## Data Availability

The four main group networks are available in Supplementary Materials, and all other intermediate data are available from the corresponding author upon request. Further, the codes for this study are available at https://github.com/Alirezafathian/fmriprediction.
