# Supplementary material for: The trend of disruption in the functional brain network topology of Alzheimer’s disease
Source: Sci Rep. 2022 Sep 2;12:14998. doi: 10.1038/s41598-022-18987-y (PMC9440254; doi:10.1038/s41598-022-18987-y)
Supplement: Supplementary file 5 — Supplementary Information 5. [file 41598_2022_18987_MOESM5_ESM.pdf]

The trend of disruption in the functional brain network topology of Alzheimer's disease

Alireza Fathian[1], Yousef Jamali[1,*], Mohammad Reza Raoufy[2], and for the Alzheimer's Disease Neuroimaging Initiative[*]

[1]Biomathematics Laboratory, Department of Applied Mathematics, School of Mathematical Science, Tarbiat Modares University, Tehran, Iran
[2]Department of Physiology, Faculty of Medical Sciences, Tarbiat Modares University, Tehran, Iran
[*]y.jamali@modares.ac.ir
[*]Data used in preparation of this article were obtained from the Alzheimer's Disease Neuroimaging Initiative (ADNI) database (adni.loni.usc.edu). As such, the investigators within the ADNI contributed to the design and implementation of ADNI and provided data but did not participate in analysis or writing of this report.

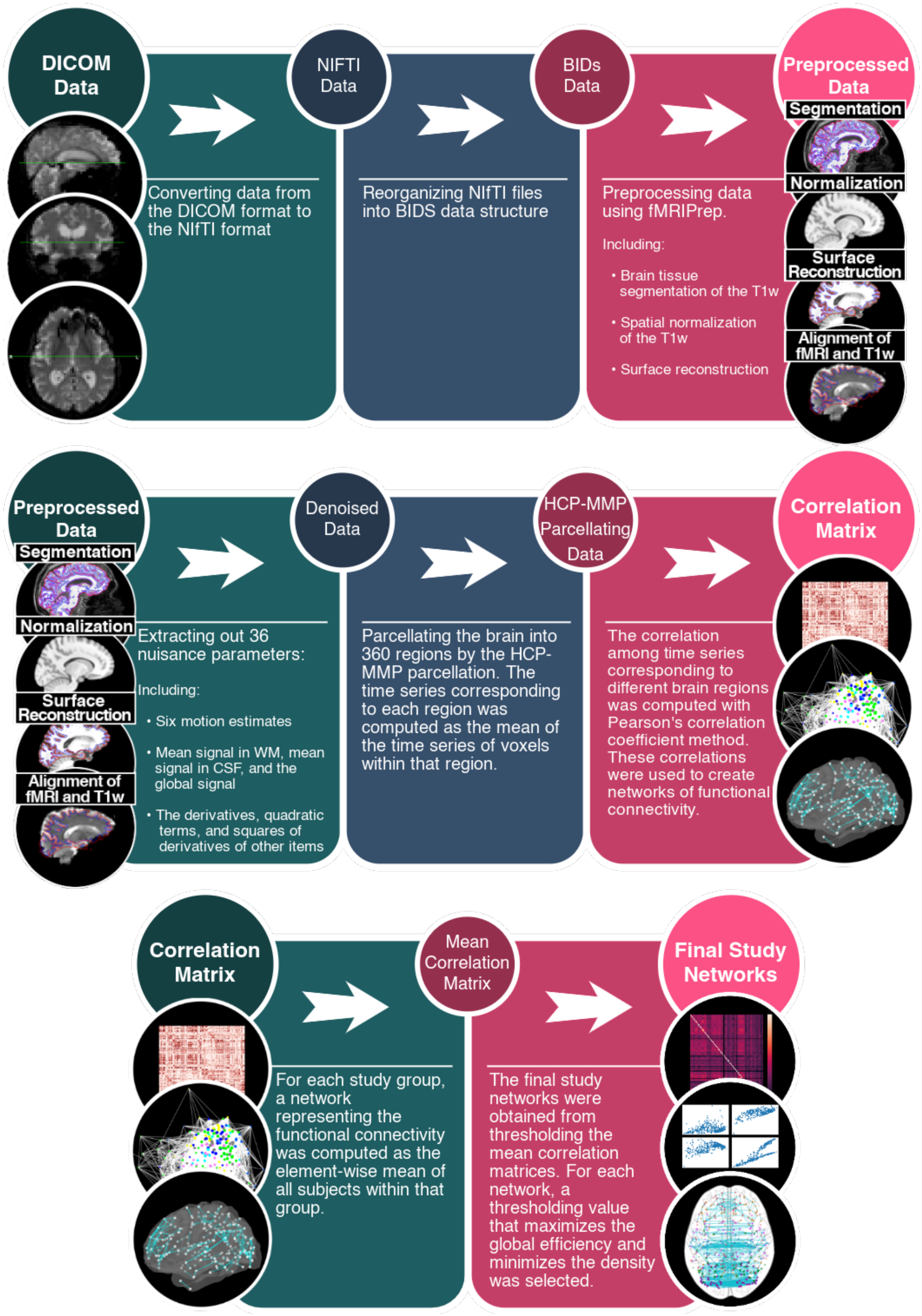

Figure S1. The overall processing pipeline of the present study. *Nilearn*, *fMRIPrep*, and *draw.io* were used to create this figure.

a

	CN	EMCI	LMCI	AD
CN	1.000	0.882	0.824	0.683
EMCI	0.882	1.000	0.840	0.710
LMCI	0.824	0.840	1.000	0.799
AD	0.683	0.710	0.799	1.000

b

	CN	EMCI	LMCI	AD
CN	0.869±0.017	0.858±0.012	0.809±0.028	0.704±0.031
EMCI	0.861±0.011	0.861±0.011	0.821±0.026	0.725±0.03
LMCI	0.807±0.03	0.819±0.027	0.813±0.016	0.775±0.024
AD	0.707±0.034	0.728±0.033	0.778±0.026	0.786±0.018

Figure S2. (a) The PCC between the four main study networks. (b) For each study group, the 23 subjects of each group were randomly divided into two subgroups of 11 and 12 networks. Then, the mean networks of these 12 and 11 subjects were constructed, and the PCC between these two mean networks was computed. This process was repeated 100 times resulting in 100 PCC matrices. This panel shows the element-wise mean and SD of these matrices.

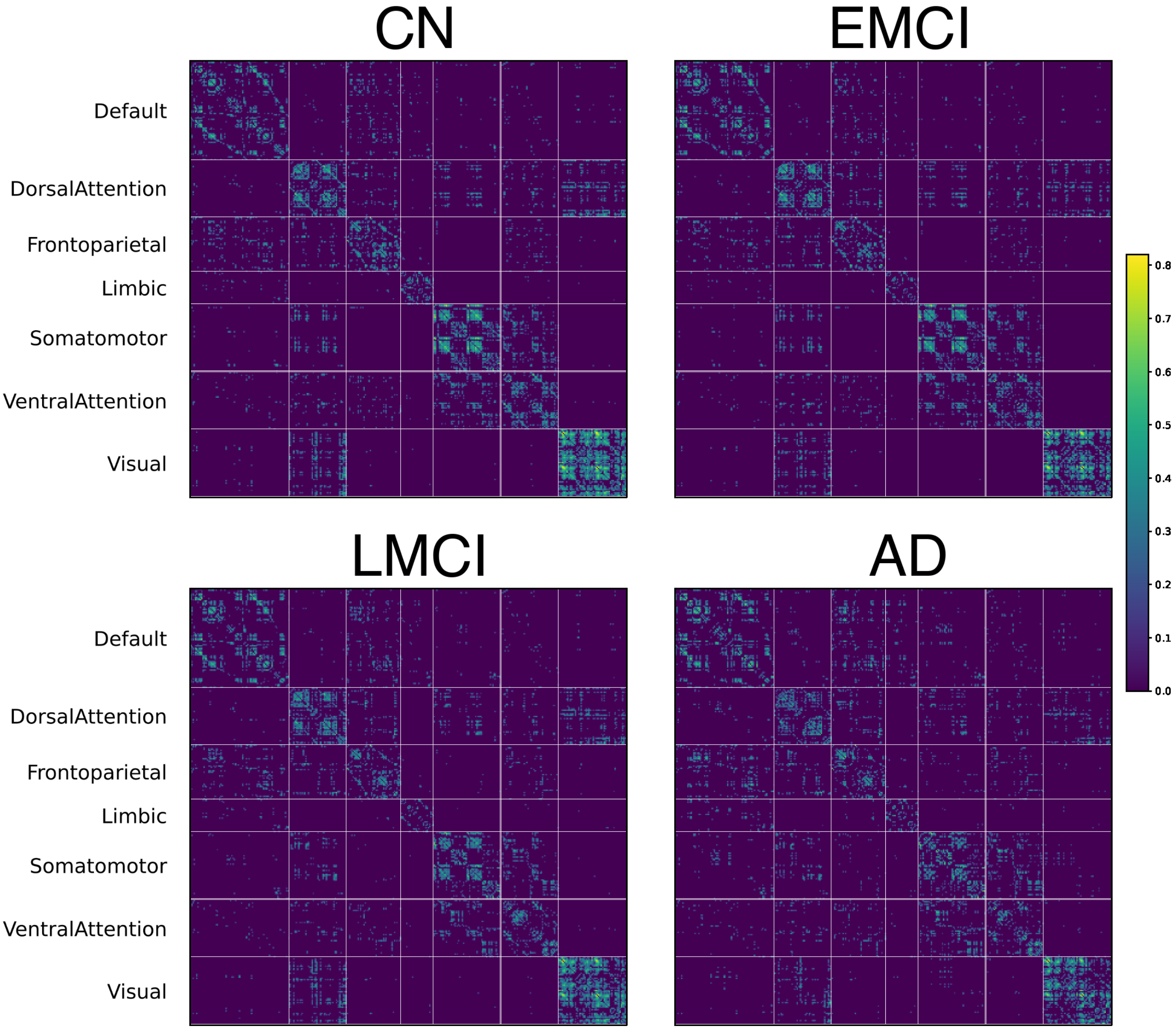

Figure S3. The correlation matrix of each study group

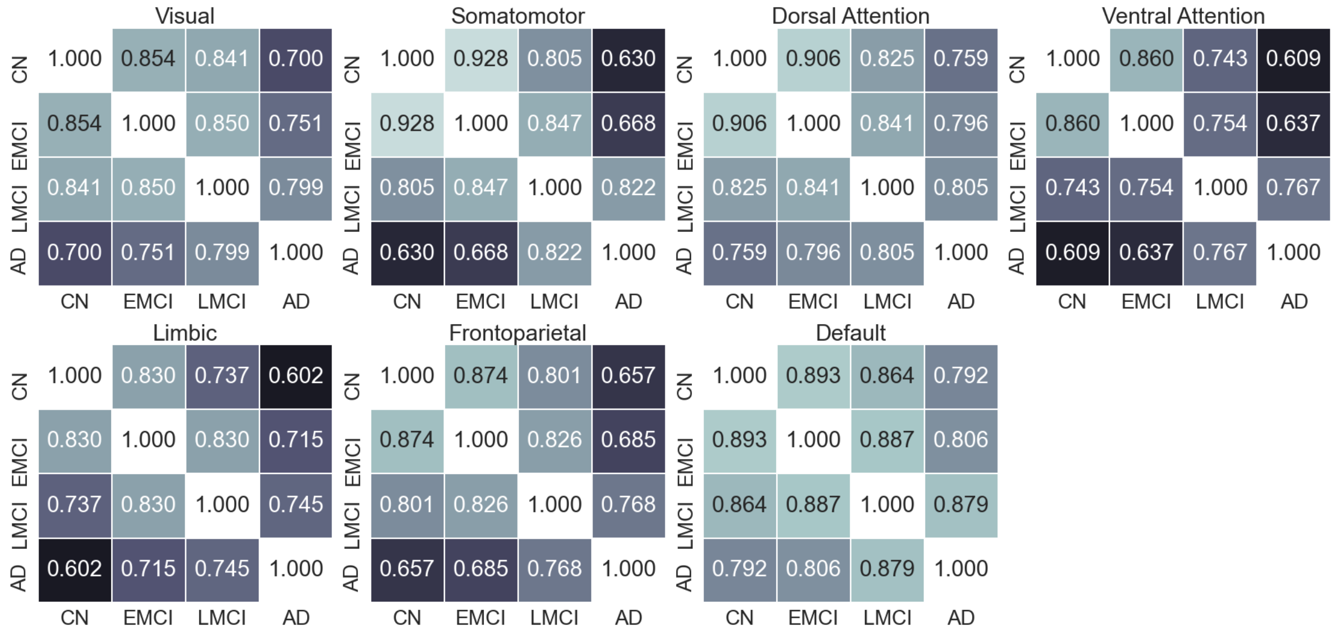

Figure S4. For each study group, the modular sub-networks containing the vertices belong to each module were computed. Then, for each module, the PCC was computed among the adjacency matrices of the modular sub-network of each study network.

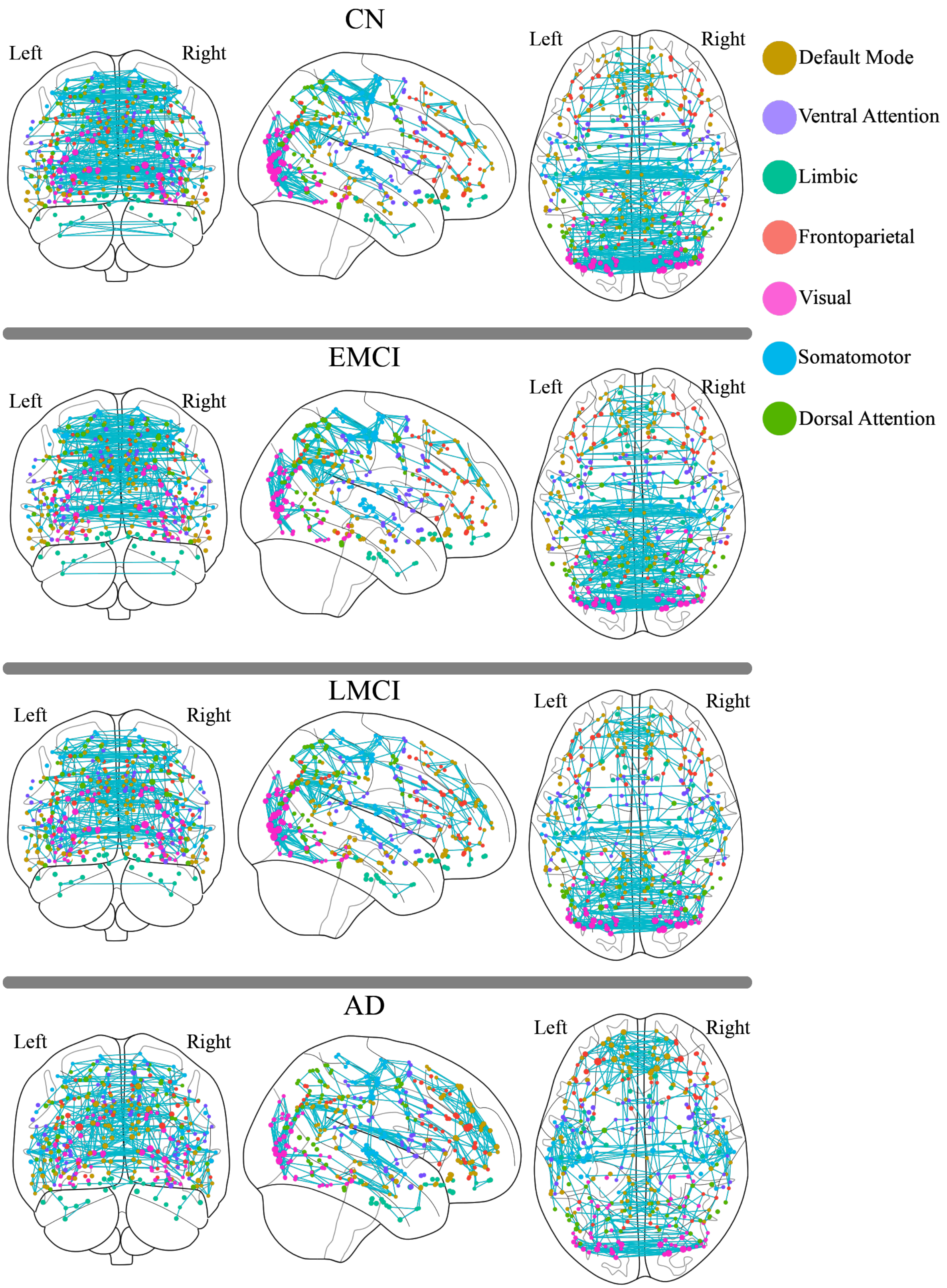

Figure S5. The top 1 percent highly-weighted links of each group network. Vertex color represents the module the vertex belongs to, and the vertex size is proportional to the vertex strength. It shows that there is a trend towards the increase in the number of default mode links as well as the decrease in the inter-hemisphere links in dorsal attention.

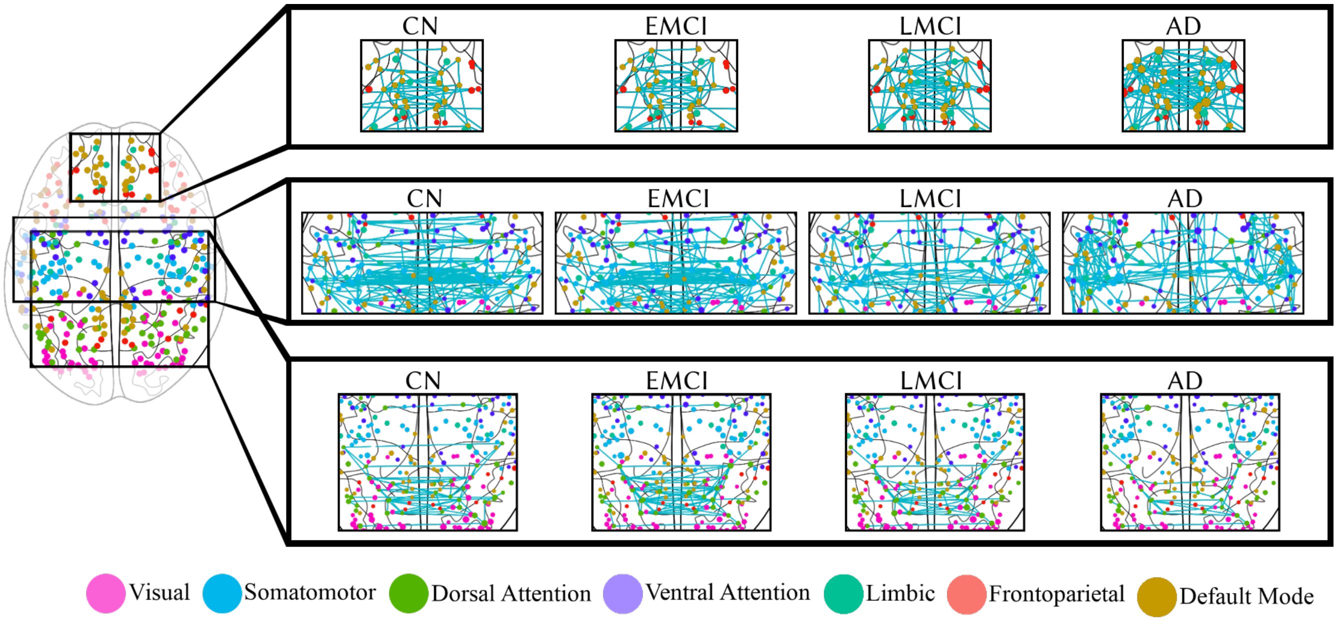

Figure S6. The axial view of the overall gradually changing patterns of top 1 percent highly-weighted links.
